# Efficacy of omega-3 fatty acids supplementation in treatment of uremic pruritus in hemodialysis patients: a double-blind randomized controlled trial

**Published:** 2012-09-30

**Authors:** E Ghanei, J Zeinali, M Borghei, M Homayouni

**Affiliations:** 1 Assistant Professor of Nephrology, Urology and Nephrology Research Center(UNRC), Shohada Medical Center, Nephrology Dept., Shahid Beheshti University, M.C.(SBMU), Tehran, I.R. Iran; 2 Resident of Internal Medicine, Shohada Medical Center, Internal Medicine Dept., SBMU, Tehran, I.R. Iran; 3 General Practitioner, UNRC, Tehran, I.R. Iran; 4 Assistant professor of Internal medicine, Department of Internal medicine, Shohada Hospital,Shahid Beheshti university of medical sciences, Tehran, I.R. Iran

**Keywords:** Fatty Acids, Omega-3, Fish Oils, pruritus, Renal Dialysis, Uremia

## Abstract

**Background:**

Uremic pruritus is a common and bothersome complaint among end-stage renal disease which affect between 25% and 60% of this population .But there is no decisive cure for treatment of it. In this study, the effects of omega-3 for treatment of pruritus were investigated in hemodialysis patients.

**Methods:**

A double-blind randomized study was carried out in the form of placebo-controlled crossover study in four dialysis centers in Tehran, Iran during 2008. At first, 22 hemodialysis patients suffering from pruritus with previous drug resistance were selected. Next, these patients were randomly allocated into two groups of omega-3-placebo (group A) and placebo-omega-3 (group B) .Patients in group A were treated with a 1-gram Fish oil capsule for 20 days, and subsequently, they were treated with placebo for 20 days after a 14-day wash-out period .But the reverse act was done in group B.The pruritus assessment was made quantitatively through Detailed Pruritus Score.

**Results:**

Pruritus was decreased up to 65% from score mean of 20.3 (95% CI: 16.7-23.8) to 6.4 (95% CI: 2.9-9.8) in omega-3 group and the decrease in the placebo group was 15% from score mean of 17.0 (95% CI: 12.4-21.6) to 14.4 (95% CI: 10.5-18.2).So the level of statistical difference was significant (P=0.0001).

**Discussion:**

Omega-3 fatty acids found to be more effective than placebo in decreasing of uremic pruritus. So it seems that Omega-3 fatty acids could be used as an efficient drug for treatment of pruritus in uremic patients.

## Introduction

One of the wearisome symptoms in the patients with end-stage renal disease (ESRD) is uremic pruritus. This complaint is observed in up to 60% of dialysis patients.(1-5) There are some factors which are known as etiology of pruritus in these patients, however, a definite etiology has not been detected yet.(1) These factors include dry skin(6,7) inadequate dialysis(8,9), anemia(10) peripheral neuropathy, (11)immune dysfunction (12), plasma level rises for histamine and chronic inflammation.(13).

On the basis of the aforesaid pathophysiologic mechanisms, several treatments have been suggested such as optimizing the dialysis dose(9), topical capsaicin(14), opioid antagonists like naltrexone, ultraviolet therapy(15), nalfurafine(16), gabapentine(17,18) erythropoiesis-stimulating agents(19), evening primrose oil(20), loratadine(21), cromolyn sodium(13,22) cholestyramine, activated charcoal(23), ketotifen(24) and gamma-linolenic acid.(25) Nonetheless, no conclusive treatment have known for this debilitating disorder yet(24). In addition, the other responsible mechanisms for uremic pruritus are increasing of the free radicals, inflammation, and leukotriene B4, immune system deficiency, and decreasing of essential fatty acids.(20) In the other hand, the amount of taken Fish oil in uremic patients is lower than the normal people for a variety of reasons such as anorexia.

Finally, because of the fish oil mechanism in reducing of inflammation, free radicals, and leukotriene B-4, plus supporting of immune system(26), this has been hypothesized that omega-3 fatty acids (fish oil) should be effective in uremic pruritus. Moreover, fish oil was found to be a safe drug which has other benefits on uremic patients such as reducing cholesterol, triglyceride, LDL, blood pressure, atherosclerosis, and oxidative stress when compared with placebo which was used in hemodialysis patients with refractory pruritus for over three months.(27) In this study, we want to assess the effects of omega-3 for treatment of pruritus in hemodialysis patients. In this study, we have investigated patients with end-stage renal disease (ESRD) who were under the intermittent hemodialysis treatment and had pruritus for over three months with no response to anti-pruritus drugs.

Pruritus has been known as a poorly localized, unpleasant sensation which lead to scratch1 The exclusion criteria were:

● Patients with the history of pruritus because of skin diseases before beginning of the renal failure, or the pruritus due to the skin diseases beyond the skin findings in the uremic patients.

● Patients with systemic diseases such as malignancies, hepatic cholestasis, hepatitis B and C, and the patients under steroid treatment.

● Patients with hemoglobin below 10g /dl and KT/V index of less than 1.2 (where K is the urea clearance (milliliters per minute); T, the treatment duration (in minutes), and V, the volume of urea distribution (milliliters) that is a useful guide for estimating the adequacy of dialysis therapy prescriptions).

● The patients under warfarin treatment and those have allergy to fish oil.

The study was performed as a randomized, double-blind, placebo-controlled crossover study on 22 patients in four dialysis centers at Shohada , Labbafinejad, Taleghani, and Chamran Hospitals in Tehran from May to September 2008. These patients were under hemodialysis three times a week, for 4 hours each session and did not have the exclusion criteria.

Informed consents were obtained from all patients who were included in the trial, and patients were able to leave the study if wanted.

Patients were divided into two groups randomly by alternation method as group A: omega-3-palcebo and group B: placebo-omega-3. There were 11 patients in group A, and 11 in group B. In group A, treatment was done with omega-3, a one-gram capsule every eight hours for 20 days (3 gram fish oil every day), and then, after a 14-day washout period, treatment continued with a one-gram placebo capsule every eight hours for another 20 days and the reverse was carried out in group B. All other anti-pruritus treatments were discontinued one week before the study. KT / V index, blood pressure, cholesterol, and triglyceride were measured before and after each study. Patients’ compliances were investigated through weekly visits and checking for the empty drug blister packs. Fish oil and placebo capsules with the same shape and volume were supplied for the patients through a pharmaceutical company (Zahravi, Tabriz, Iran). The used fish oil contained 30% long-chain n-3 polyunsaturated fatty acids as one-gram capsule that contained 180 mg of Eicosapentaenoic Acid (EPA) and 120 mg of Docosahexaenoic Acid (DHA); however, the one-gram placebo lacked any EPA or DHA but contained long chain omega 3 fatty acids Pruritus assessment: The pruritus assessment was carried out throughout the study by the same person at the start, during, and at the end of the study. To assess the pruritus, Detailed Pruritus Score method devised by Dr. Duo(28), was utilized in our study. In this method, intensity, distribution, and the frequency of pruritus that had led to sleep disturbance was scored according to the following. The collection and the data gathering technique were based on observation and interview. The data for this questionnaire were collected from the patients. We assess patients in weekly visit at dialysis center for side effect but the pruritis score was calculated before and after consuming placebo and omega 3.

Severity: sense of itching with no need to scratch received 1 point; few time of scratch without excoriation 2 point; frequent need to scratching 3 points; scratching with excoriation 4 points, and an itch that lead to continuous unrest 5 points. (A maximum of 10 points can be calculated during the day (5 in the morning,5 in the afternoon)

Distribution: 1 point for pruritus in less than two areas; 2 points for pruritus in more than two areas; and 3 points for a widespread pruritus. The recorded scores for intensity and distribution were multiplied separately for morning and the afternoon. The maximum score was 30 points.

Sleep disturbance: 2 points for each wakeup because of pruritus (with a maximum of 10 points); and 1 point for each scratching with excoriation during the night (with a maximum of 5 points). Sleep disturbance and intensity-distribution scores were added up to calculate the patient’s final score at the start and at the end of the study.

Severity score was multiplied to Disturbance for morning and afternoon separately. Then morning score add to afternoon score. finally this score has added to Sleep Disturbance.

We calculated the mean of score among 22 patients before and after using omega 3 besides before and after using placebo. After that the variation of this score after using omega 3 and placebo was calculated. Finally these two variation (after using placebo and omega 3 were compared.

The ethics involved in this study was approved by the research deputy office of the internal medicine group at Shahid Beheshti Medical University.

Before starting of the project, the study was explained to the patients and they were entered in the study voluntarily, and patients underwent the experiment after writing consent. Considering the safety of the prescribed drug and its other benefits, its rare complications such as dyspepsia, skin rash, headache were explained to the patients that if they occurred, could mandate the drug to be discontinued and reported, otherwise, the drug’s prescription had no ethical prohibitions. Because of no proper response to anti-pruritus drug, the discontinuation of this drug and using placebo instead did not seem to be unethical.

### Statistical analysis

The obtained results had been recorded as Detailed Pruritus Score, and were presented as percent, mean, and 95% confidence interval. To analyze the statistics, T –student (paired T-test) test was utilized that a P value of less than 0.05 was considered as significant. Chi Square Test was used to analyze the qualitative factors.

The pruritus assessment results were computed quantitatively by using Detailed Pruritus Score table, and then, were analyzed statistically by computer. To analyze and determine the significance of the results, independent sample t test was used.

**Fig 1:**
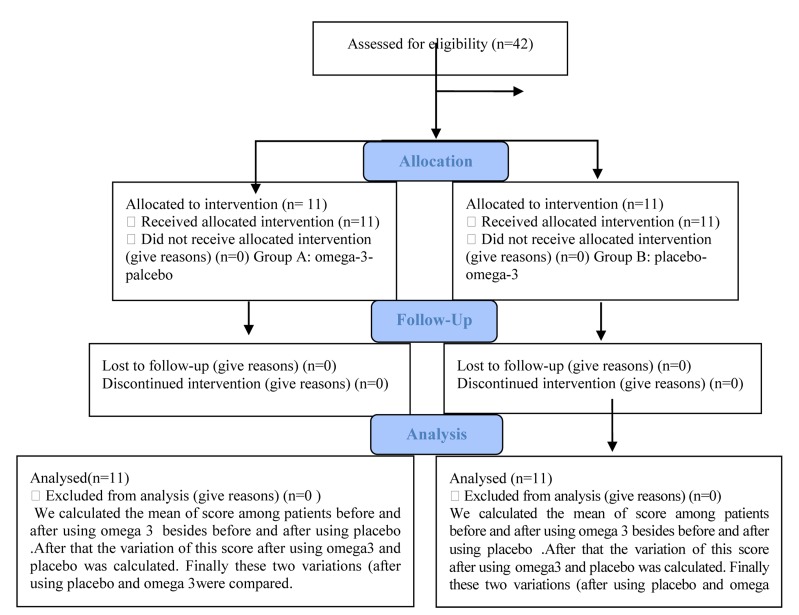
Flow Diagram

The ethical aspects of the project:

Progress through the phases of a parallel randomised trial of two groups are shown in a flow diagram (Fig 1).

22 patients, 14 male and 8 female, in the age range of 20 to 85 years old were entered in this study. The mean age was 55 years old for men and 58 years old for women. The characteristics of all patients are shown in (Table 1).

**Table 1:** The characteristics (mean± SD) of hemodialysis patients with pruritus

Groups	age	sex	Duration of HD (years)	Time of HD (h)	EPO	ALP (units/L)	Hb (g/dL)	Ca (mg/dL)	P (mg/dL)	Cr (mg/dL)	Kt/V
A	59.90±14.82	72% Men 28% Women	3.81±2.04	4±0	+	339.9±203.3	10.90±1.0	9.18±2.09	5.71±1.66	9.42±1.9417	1.24±0.05
B	53.09±13.08	54% Men 46% Women	5.09±4.88	4±0	+	402.4±237.1	9.09±1.39	9.09±1.39	5.64±1.64	10.96±5.90	1.41±0.27

All patients had KT / V index of 1.2 or above. The dialysis duration varied from 1 year to 17 years. The hemoglobin level in all patients was 10 grams / dL or higher, and all patients wouldreceive erythropoietin 2,000 units three times a week subcutaneously. The mean blood pressure in the patients was 130 / 80 mmHg before the treatment, and 120 / 80 after the treatment that its reduction was insignificant. The mean triglyceride level was 123 mg / dL before receiving docosahexaenoic (omega-3) and 126 mg/ dL after the treatment. The mean serum cholesterol was 147 mg / dL before the treatment and 135 mg /dL after it and the difference was not significant.

**Fig 2:**
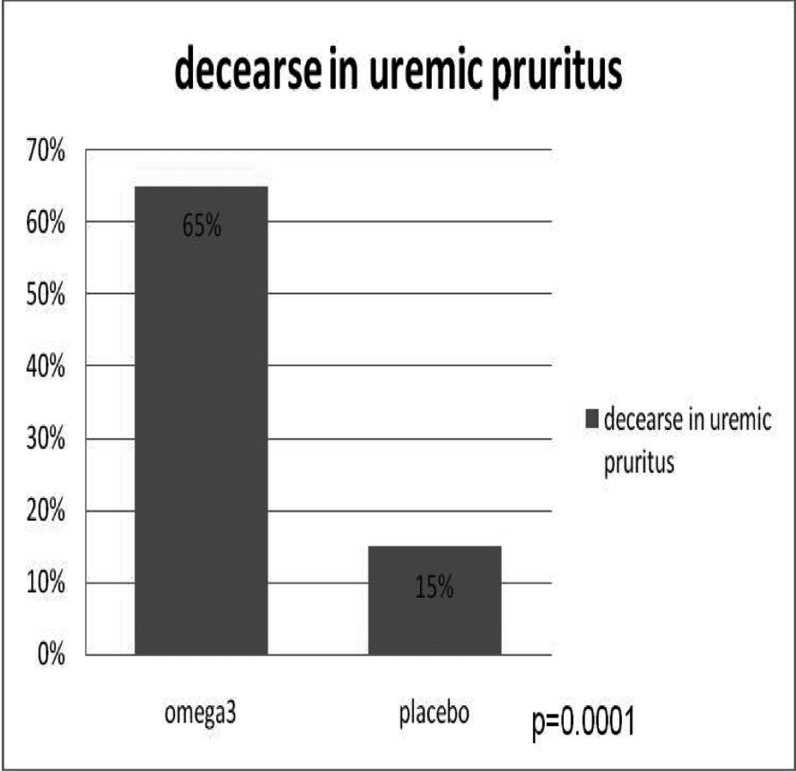
Comparison between omega-3 and placebo in uremic pruritus in 22 patients

The treatment with omega-3 reduced the pruritus in up to 65% from mean score of 20.3 (95% CI: 16.7 –23.8) to 6.4 (95% CI: 2.9 – 9.8), and in the placebo group, pruritus was reduced in up to 15% from 17 (95% CI: 12.4 –21.6) to 14.4 (95% CI: 10.5 –18.2) (Fig 2).

The statistical difference was significant (P=0.0001). So, fish oil was more effective than placebo in reducing uremic pruritus. No complications such as dyspepsia, skin rash, and headache were observed in patients.

## Discussion

In our study, the effect of omega-3 fatty acids (fish oil) on pruritus in hemodialysis patients was examined and found to be effective. In addition, Fatty acid was well-tolerated with a dose of 3 grams per day without any complications; however, no significant differences could materialize concerning the triglyceride, cholesterol, and the blood pressure of the studied patients and it was perhaps due to the short-term treatment of only 20 days.

For over a century, uremic pruritus has been recognized as a dire experience that has negative repercussions in End Stage Renal Disease patients. Up to now, the pathophysiological basis of the disease has not been understood and various pathways and mechanisms seem to be involved .So a number of hypotheses have been presented for that. To treat with this condition, many methods have been tried with contradictory and mixed outcomes(13).

One hypothesis is the fact that a misbalance in plasma composition for essential fatty acids (EFAs) is probably the underlying cause for this type of pruritus in hemodialysis patients(20, 29). Fish oil, with its eicosapentaenoic acid (EPA) and decosahexaenoic acid (DHA), is considered as a source of omega-3 fatty acid. Naturally, these fatty acids are synthesized in body, but the amount seems to be inadequate in many individuals specially in uremic patients(20).

In an effort to study the possible effects of different types of oil on pruritus symptoms, a randomized, double blind study was performed. In this study twenty-five patients were involved and randomly divided into three groups, and given 6 g of olive oil (OO), 6 g of fish oil (FO) or 6 g of safflower oil (SO) for 8 weeks. Patients were evaluated for their pruritic symptoms (in terms of severity, distribution, and frequency) at first and at 8 weeks. At the end of the study, the scores for severity and distribution of pruritus symptoms have declined more in the FO group when compared with the other two groups (10>P>0.05). The researchers Construed that the improvement in symptoms was caused by a falling in leukotriene B4 (LTB4; a proinflammatory eicosanoid), but LTB4 was not calculated(30).

Another Randomized, prospective, double-blind study was done to assess the effect of supplementation with different sources of oils rich include fish oil (FO) and safflower oil (SO), on the raising of leukotriene B4 (LTB4) by polymorphonuclear leukocytes (PMNLs) in patients under hemodialysis treatment and the consequent effects on the symptoms of pruritus .In this study ,twenty-two patients on maintenance hemodialysis were involved with complaint of itching and divided to two groups receiving daily supplements of 6 g ethyl ester of FO or SO for 16 weeks. As the result, the change in LTB4 production ``g/mL) from baseline to the end of study was 240.7 +/- 200.2 to 29.2 +/- 14.6 in the FO group and from 171.1 +/- 121.7 to 31.9 +/- 14.7 in the SO group. The overall pruritus score was changed from 16.7 +/- 11.4 to 8.9 +/- 9.2 in the FO group and from 17.5 +/- 8.8 to 13.1 +/- 5.6 in the SO group. So, FO supplementation did not cause a significant specific effect on producing of LTB4 by the PMNLs but the percent decline in total puritus score was greater for the FO group in comparison with the SO group(26).

Fish oils, EPA and DHA have a pivotal role in production, and therefore, maintaining the normal function of the nerve tissue and cellular membranes(31,32). Series-3 prostaglandins are formed through conversion of EPA that possess anti-inflammatory effects(33,34). The other roles of these fats are reducing the high blood pressure, the cholesterol and triglyceride rise(33). They could also prevent the formation of the atherosclerotic plaques, and could relieve skin disease such as eczema (35) and even psoriasis(36).

The most common complication after taking fish oil is gastrointestinal disturbances including nausea which occurs in 4% of the patients in doses of 3 grams per day or above(37). The use of Fish oil in doses of over 3-4 grams per day reduces the serum triglyceride concentration to 25-30%(38,39).

Fish oil supplements with an average dose of 3.7 g /d and median duration of eight weeks found to be effective for decreasing the systolic and diastolic blood pressures by 3.5 mmHg and 2.4 mmHg in adults with ages above 45 in a meta-analysis study(40).

No study has succeeded in proving the link between the fish oil intake and the C-reactive protein levels(39,41). On the contrary, it seems that fish oil supplements are able to inhibit production of cytokines such as interleukin-1 beta and the tumor necrosis factor alpha(42). To observe such results, however, relatively high doses of fish oil (above 2 grams per day) appear to be necessary. Many other randomized trials have been demonstrative of the lowering effects of fish oil on the circulating endothelial dysfunction markers among them E-Selectin, vascular cell adhesion molecule-1 (VCAM-1), also the intracellular adhesion molecule –1 (ICAM-1),(41,43).

Fallahzadeh et all showed that serum levels of IL-2 were significantly higher in HD patients with itch in comparing with those without it(44). In the other hand, the Th1 effector cell proliferative response to antigen was decreased in the fish oil group and fish oil can mitigate the effect of interleukin 2(45). So it can be considered as another mechanism for fish oil.

In this study, though the aforesaid effects were not studied, yet due to the inflammation increase in hemodialysis patients, the long-term intake of fish oil could be potentially useful in this aspect.

Considering the prevalence of pruritus in uremic patients and a lack of decisive treatment for it, accompanied by the abnormal essential fatty acids pattern in the uremic patients and their role in the pathogenesis of the uremic pruritus, fish oil supplements could improve the uremic pruritus.
